# MorphoGrad: A MATLAB toolbox for simulating steady-state morphogen gradients under cell-to-cell variability

**DOI:** 10.1016/j.xpro.2026.104611

**Published:** 2026-06-03

**Authors:** Jan A. Adelmann, Roman Vetter, Dagmar Iber

**Affiliations:** 1 Department of Biosystems Science and Engineering, ETH Zürich, Schanzenstrasse 44, 4056 Basel, Switzerland; 2 Swiss Institute of Bioinformatics, Schanzenstrasse 44, 4056 Basel, Switzerland

**Keywords:** Bioinformatics, Biophysics, Developmental biology, Systems biology, Physics

## Abstract

Studying morphogen gradient-based tissue patterning *in silico* while accounting for biological variability remains challenging. Here, we present a MATLAB-based protocol for simulating steady-state morphogen gradients in one- and two-dimensional tissues with cell-to-cell variability in model parameters. We describe steps for configuring cell-based geometries, defining reaction-diffusion equations and model parameters, assigning stochastic parameter variability, setting boundary conditions and solver options, running simulations, and analyzing stochastic gradients to quantify patterning precision.

For complete details on the use and execution of this protocol, please refer to Long et al.^1^

## Highlights

• Instructions for configuring one- and two-dimensional simulation geometries
• Steps for defining reaction-diffusion models and stochastic parameters
• Guidance on selecting initial guesses, boundary conditions, and solver settings
• Procedures for analyzing stochastic gradients and patterning precision

## Abstract (editor highlights)

Publisher’s note: Undertaking any experimental protocol requires adherence to local institutional guidelines for laboratory safety and ethics.

## Graphical abstract

**Figure:**
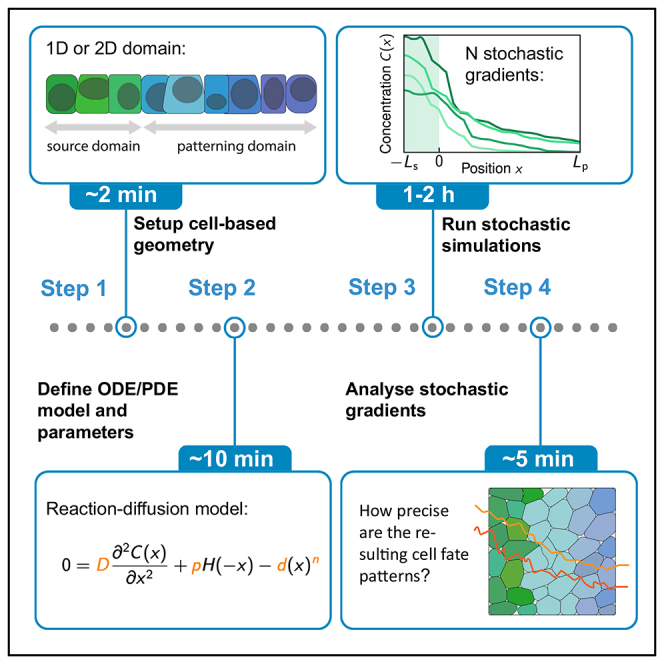

## Before you begin

This protocol describes how to generate and analyze synthetic morphogen gradients under cell-to-cell parameter variability in one- and two-dimensional epithelial tissues using MATLAB, based on the conceptual framework introduced by Vetter and Iber.^2^ In this model, morphogen molecules are produced within a designated source region and then diffuse across the surrounding tissue, where they bind to receptors, degrade, or get turned over otherwise. To represent this process mathematically, we solve steady-state reaction-diffusion equations of the form:

DΔC(x,y)=R(C,x,y)

where *C*(*x*,*y*) is the morphogen concentration at position (*x*,*y*) in the tissue, *D* is the morphogen diffusion coefficient, Δ is the Laplace operator, and *R*(*C*,*x*,*y*) represents parameterized chemical reaction terms. In previous applications, we used the following form:

$$D\left(\frac{\partial^{2}c\left(x,y\right)}{{\partial x}^{2}}+\alpha \frac{\partial^{2}c\left(x,y\right)}{{\partial y}^{2}}\right)=dC{\left(x,y\right)}^{n}-pH\left(-x\right)$$

Here, *α* is an anisotropy factor that allows diffusion to differ between the *x*- and *y*-directions. The term *dC*(*x*,*y*)^*n*^ models concentration-dependent degradation at rate *d* with an exponent *n*, where values *n*>1 model nonlinear decay.^3^ *pH*(-*x*) represents morphogen production restricted to the source region, with *H* the Heaviside step function, which evaluates to 1 for positive arguments (i.e., inside the source) and to 0 for negative arguments (i.e., outside the source). For one-dimensional simulations, the *y*-dependence and the corresponding diffusion term are removed.

The computational framework divides both the source and the patterning region into discrete cells. Each cell can have its own parameter set (for example, diffusion, degradation, or production rates), enabling simulation of tissue-level heterogeneity. To incorporate biological variability, parameters are sampled from log-normal distributions. Log-normal distributions are biologically plausible, as many measured cellular properties, such as cell areas and biochemical rate parameters, are right-skewed and strictly non-negative. As shown in Adelmann et al.,^4^ the precise choice of distribution does not affect the qualitative behavior of the simulated gradients, if certain properties (such as non-negativity) are met.

Morphogen gradients play a central role in many developmental processes by providing positional information that enables cells to adopt distinct cell fates within a tissue. According to Wolpert’s famous French flag model, fixed thresholds *C*_*θ*_(*x*_*θ*_,*y*) define the position of domain boundaries.^5^ Due to biological variability, boundary positions vary between embryos *i*, resulting in a positional error that can be quantified as the standard deviation of the readout positions,

$$\sigma_{x}=std\left(x_{\theta ,i}\right)\text{.}$$

**Figure 1:**
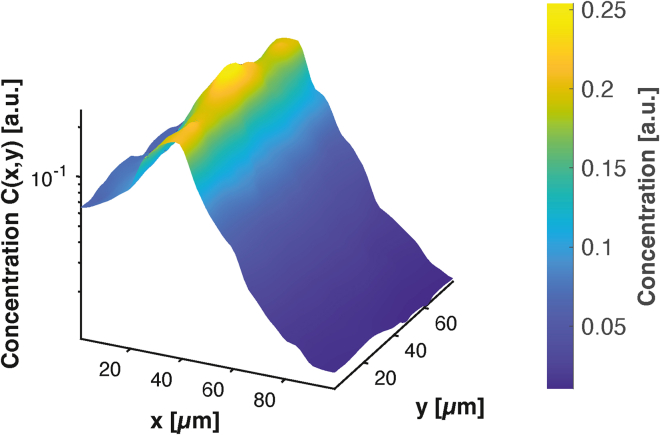
Visualization of a 2D stochastic gradient simulation output

Quantification of the positional error allows us to assess how cell-to-cell parameter variability and cell-area variability affect precision of cell fate boundaries.^6^ An example output of a noisy two-dimensional gradient is shown in Figure 1.

### Innovation

MorphoGrad provides a computational framework for simulating steady-state morphogen gradients on a cellular domain while explicitly incorporating cell-to-cell parameter variability. The framework features a graphical user interface (GUI) that allows users to configure simulations without modifying the source code. Simulations can be configured entirely through the GUI, allowing users to focus on biological modeling rather than numerical implementation.

MorphoGrad supports simulation in both one- and two-dimensional tissues, enabling direct investigation of how variability in kinetic parameters affects patterning precision. Built-in analysis utilities facilitate the extraction of key statistical measurements from ensembles of stochastic gradients. While ensemble simulations require minimal user scripting to execute repeated solver runs, the underlying numerical solver and analysis routines are provided as part of the framework and do not need to be modified by the users. This lowers the barrier to performing reproducible stochastic morphogen gradient simulations.

### Download and install the MorphoGrad toolbox

**Timing: 1–10 min**

1. Install MATLAB R2022a or later. If the user wishes to parallelize simulations, ensure that the Parallel Computing Toolbox is installed.
2. Download the MorphoGrad code using one of the following methods:
  a. Using GitOpen a UNIX terminal and run: git clone git@git.bsse.ethz.ch:iber/Publications/2026_adelmann_morphograd.git. This creates a local folder named morphograd containing the toolbox.
  b. Using a zip fileDownload the.zip archive from: https://git.bsse.ethz.ch/iber/Publications/2026_adelmann_morphograd and extract it to a convenient location.
3. Run the setup script. In MATLAB, change into the folder containing setup.m and the +morphograd directory. Run the setup.m script, which adds the toolbox folder to the MATLAB search path.
4. Launch the graphical configuration builder to set up simulations:
  morphograd.gui.ConfigBuilder()

## Key resources table

**Table:** 

REAGENT or RESOURCE	SOURCE	IDENTIFIER
**Software and Algorithms**		

MATLAB R2022a or newer	The MathWorks, Inc.	https://www.mathworks.com
MATLAB files for simulation	This work	https://git.bsse.ethz.ch/iber/Publications/2026_adelmann_morphograd https://doi.org/10.5281/zenodo.20134353

## Step-by-step method details

### Setting up the geometry and domain

**Timing: 2–5 min**

In this section, we describe how to configure the simulation geometry and domain using the MorphoGrad graphical user interface (GUI).

1. Launch the GUI by calling morphograd.gui.ConfigBuilder().
2. In the Setup tab, select whether the simulation is one- or two-dimensional (Figure 2).

**Figure 2:**
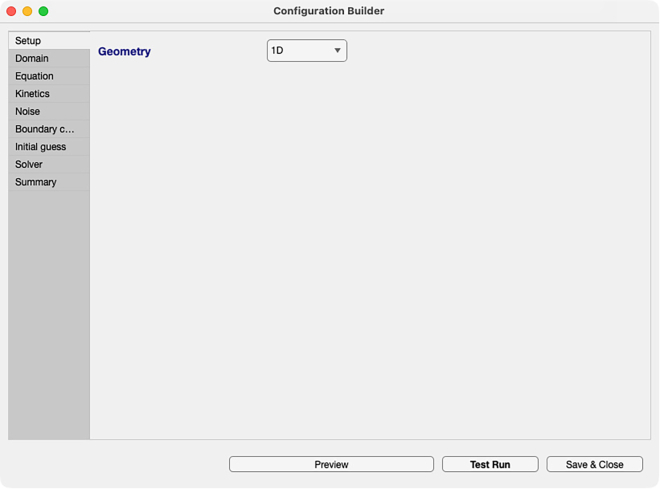
Defining the dimension in the configuration builder
3. Open the Domain tab (Figure 3).
  a. 1D: Define the total domain length *L*. Specify the start and end positions of the source region. The remaining domain is labeled as patterning domain.
  b. 2D: Additionally specify the tissue width in *y*-direction. Specify distinct diameters in *x*- and *y*-direction.

**Figure 3:**
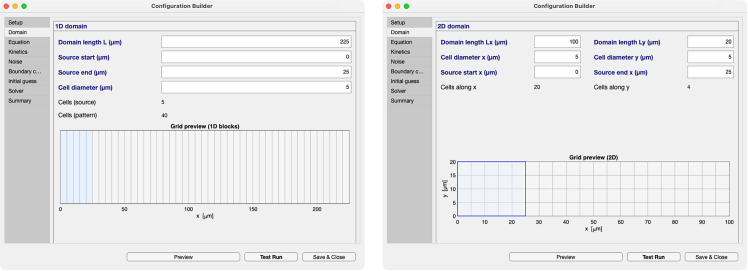
Setup of 1D and 2D domains

***Note:*** The source can be placed at an arbitrary position along the *x*-direction but always completely spans the *y*-direction.

**CRITICAL:** The patterning length, the source length, and (in 2D) the tissue width must be integer multiples of the specified cell diameters, to allow to discretize the domain into a whole number of cells. Cells are approximated as line segments in 1D and as rectangles in 2D. See troubleshooting 1.

### Setting up the differential equations

**Timing: 10–20 min**

In this section, we describe how to select preset equations or define a custom reaction term using valid MATLAB syntax.

4. Open the Equation tab (Figure 4).

**Figure 4:**
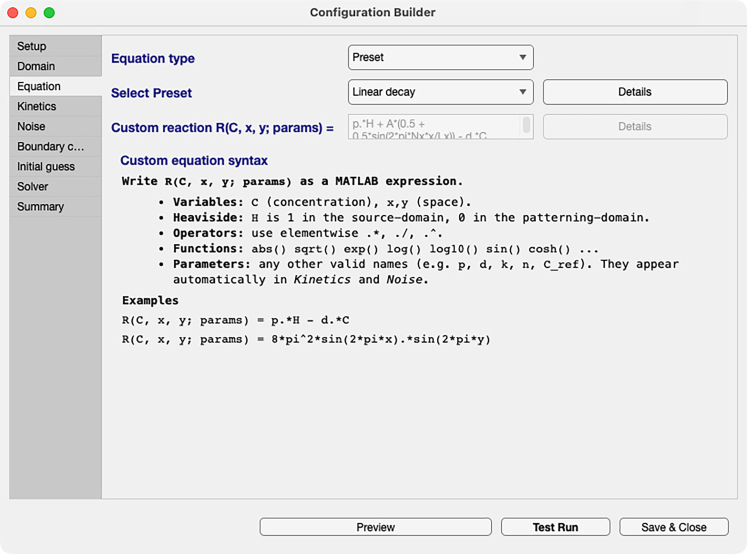
Defining the reaction terms
5. Choose whether to use a preset equation or define a custom reaction equation. The following presets are available (1D and 2D):
  a. Linear decay with production in the source and degradation:
    DΔC+pH(x)−dC=0
  b. Nonlinear decay with production in the source and nonlinear degradation (n>1):

$$D\Delta C+pH\left(x\right)-dC^{n}=0$$

6. If the presets do not match your desired model, define a custom reaction term. Enter a valid MATLAB expression in the text field. The syntax follows the following rules:
  a. Variables: *C* is the species concentration, *x* and *y* are spatial coordinates. The species can be written as *C*,*C*(*x*),*C*(*x*,*y*).
  b. Source terms: Terms active only in the source region must be multiplied by *H*, the Heaviside function which evaluates to 1 in the source and to 0 outside of it.
  c. Vectorization: The code is vectorized. If you are familiar with the syntax, use element-wise operators (.∗, ./, .ˆ).
  d. Functions: You can include standard MATLAB functions (e.g., sqrt, exp, log, log10, sin, cosh).
  e. Reserved names: Parameter names must not conflict with the reserved identifiers *C*,*x*,*y*,*D*,*α*,*n*,*H*, and *C*_*ref*.

***Note:*** The reaction term is everything excluding the diffusion term *D*Δ*C* and has the form *R*(*C*,*x*,*y*;*params*), where *C* is the species (e.g. the morphogen) concentration and *params* are the different parameters.

**CRITICAL:** Highly nonlinear equations (*n*≫1) can lead to slow solver convergence. In such cases, provide an appropriate initial guess or adjust solver error tolerance as needed.

### Defining model parameters

**Timing: 2–10 min**

In this step, model parameters governing transport and reaction kinetics are specified. These parameters define, for example, diffusivity of the morphogen, production and degradation rates, and any other specified kinetic constants.

**Figure 5:**
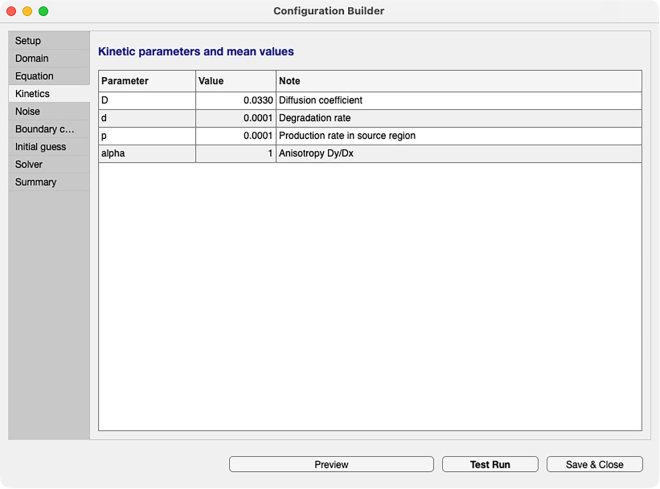
Defining the kinetic parameters of the model
8. Specify numerical values for each parameter in the differential equation, including the diffusion coefficient *D*.

***Note:*** In the two-dimensional setting, an additional parameter *α* controls diffusion anisotropy. This parameter can take values in the range [0, 1], where *α*=0 corresponds to no diffusion in *y*-direction and *α*=1 corresponds to isotropic diffusion. The 2D diffusion operator changes to:

7. Open the Kinetics tab (Figure 5).

$$D\left(\frac{\partial^{2}C\left(x,y\right)}{{\partial x}^{2}}+\alpha \frac{\partial^{2}C\left(x,y\right)}{{\partial y}^{2}}\right)$$

**CRITICAL:** Solver convergence strongly depends on the parameter regime, particularly for nonlinear reaction terms. Inappropriate combinations of production, degradation, and nonlinearity may lead to stiff or unstable systems. If the solver does not converge, first reassess the chosen parameters.

### Defining noise levels

**Timing: 2–10 min**

In this step, we describe how stochastic parameter variability is introduced into the model. Each simulation corresponds to one realization in which selected parameters are sampled independently, for each cell from log-normal distributions with user-defined mean values and coefficients of variation (CVs).

9. Open the Noise tab (Figure 6).

**Figure 6:**
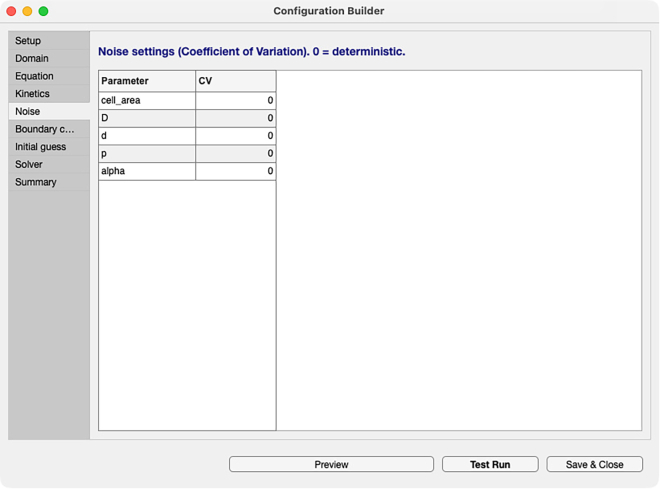
Defining kinetic noise levels
10. To introduce parameter variability, specify a coefficient of variation (CV) greater than zero for any parameter. A value of 0 disables stochasticity in the corresponding parameter.

***Optional:*** Variability in cell areas can be introduced by setting a CV for the cell area.

***Optional:*** In two dimensions, stochasticity in the anisotropy *α* can be specified.

***Note:*** The magnitude of the noise is controlled by the coefficient of variation (CV), defined as the ratio of the standard deviation to the mean (CV = *σ*/*μ*). Smaller CV values result in parameter values tightly clustered around the mean. Parameters are sampled independently from log-normal distributions, ensuring non-negative values, for each cell.

***Note:*** The framework captures static cell-to-cell variability as it arises from molecular noise. The current implementation does not simulate time-dependent stochastic reaction or diffusion events.

### Defining boundary conditions

**Timing: 2–10 min**

Here, we describe how to define boundary conditions. Boundary conditions represent assumptions on how the morphogen is exchanged with the surrounding environment at the tissue borders.

11. Open the Boundary condition tab (Figure 7).

**Figure 7:**
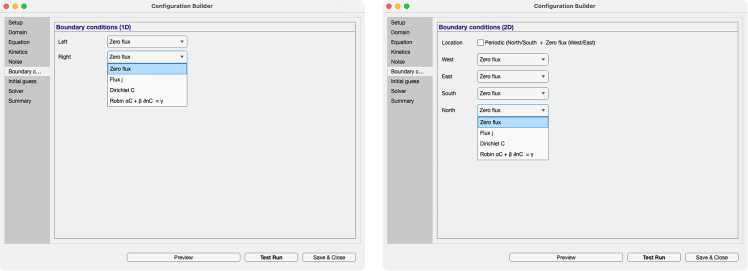
Setting boundary conditions in 1D and 2D
12. For each boundary, select from the following available boundary conditions:
  a. Dirichlet: fixed concentration value at the boundary. This boundary condition should be chosen if there is an external reservoir or sink that maintains a prescribed morphogen concentration at the edge of the simulated domain. For example, an idealized, perfectly absorbing boundary can be represented by a homogeneous Dirichlet boundary condition (*C*=0).
  b. Neumann: fixed diffusive flux across the boundary. The special case of zero-flux corresponds to an impermeable tissue edge across which no morphogen transport occurs. Non-zero Neumann conditions can be applied to impose a constant in- or outflux across a boundary.
  c. Robin: mixed boundary condition of the form
    $$\alpha C+\beta \frac{\partial C}{\partial x}=\gamma$$
    evaluated at the domain boundary. Robin boundary conditions are useful when the exchange at the boundary depends on the local concentration. For example, boundary-mediated leakage or clearance into the surrounding domain can be modeled by -*DdC*/*dx* = *kC*, corresponding to a morphogen outflux proportional to the concentration at the tissue border.
    ***Note:*** In the one-dimensional case, boundary conditions can be specified at the left and right boundary of the domain. In the two-dimensional case, boundary conditions must be specified at the west/east/south and north boundaries, in this order. Additionally, periodic boundary conditions can be applied along the north and south boundaries to mimic an infinite stripe, in that case the east and west boundary conditions are zero-flux.
    ***Note:*** The default boundary condition type is zero-flux at all boundaries.
    ***Note:*** Neumann boundary conditions with flux *γ* = -*DdC*/*dx* are recovered by setting *α* = 0 and *β* = 1 and multiplying by -*D*.

### Defining an initial guess

**Timing: 2–5 min**

In this step, we describe how to define an initial guess. The initial guess provides a numerical starting point for the steady-state solver and primarily serves to improve solver convergence and robustness.

**Figure 8:**
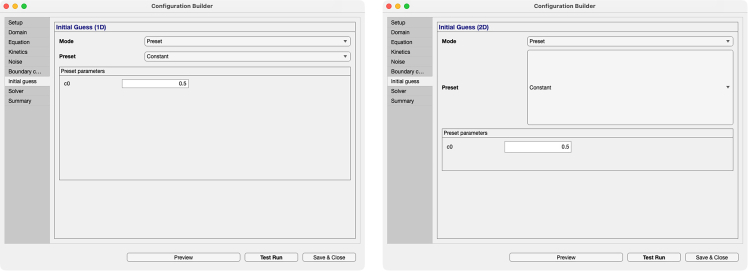
Example of defining a custom expression in 1D and using a preset initial guess in 2D
14. Select either a preset initial guess or define a custom initial guess. If no initial guess is provided, a uniform default value of 0.5 is used.

***Note:*** The default value of 0.5 was chosen due to good convergence with all preset equations.

13. Open the Initial guess tab (Figure 8).

15. In the one-dimensional setting, specify both the concentration profile and its first spatial derivative:
  C(x) = <expression>,
  C’(x) = <derivative of expression>.

***Note:*** The expressions are evaluated on the spatial mesh and passed to the boundary value problem solver (bvp4c).

16. In the two-dimensional case, specify a custom initial guess of the form C(x,y) = <expression>, with no derivative required.

***Note:*** For equations with unique steady-state solution, different reasonable initial guesses should converge to the same solution. For strongly nonlinear models, the chosen initial guess may affect convergence behavior. In cases with multiple steady states, the guess can influence which steady-state solution is reached.

***Note:*** The framework solves for steady-state solutions, therefore the initial guess often does not have a direct biological interpretation and primarily serves as a numerical starting point for the solver. When biologically motivated initial concentrations are known, they can be used as the initial guess. For example, a zero-concentration profile can be specified if there is no morphogen present to begin with.

**CRITICAL:** Define the custom initial guess using only numerical values. Do not reference parameter names (e.g., *p*,*D*,*d*). Restrict expression to the spatial coordinate *x* in 1D or *x*,*y* in 2D and to built-in MATLAB functions (e.g. exp, sin, cosh, sqrt, log), including the Heaviside function *H*. Ensure that the expression can be evaluated directly on the mesh without accessing any user-defined parameters.

### Specifying solver settings

**Timing: 2–5 min**

In this step, we describe how to adjust solver settings to control numerical accuracy. These settings control how the steady-state equations are solved on the numerical mesh. They do not change the biological model itself, but define the spatial resolution, stopping criteria, numerical accuracy, and runtime of the solver. In general, finer meshes and tighter tolerances improve numerical accuracy, but increase memory usage and computation time. For 1D problems, see substeps 18–19, for 2D problems, see substeps 20–22.

**Figure 9:**
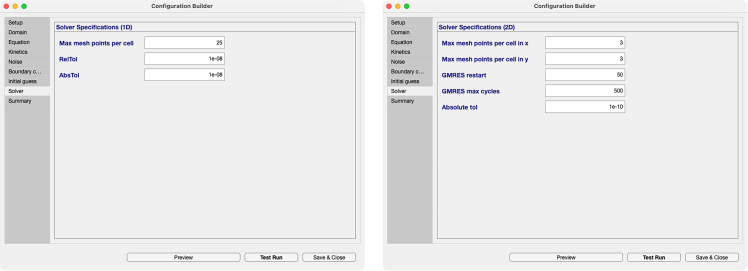
Solver settings in 1D and 2D. In the 2D case, the GMRES solver settings are shown
18. In 1D specify the maximum number of mesh points allowed during adaptive mesh refinement. This parameter sets the upper limit on how finely the solver may refine the spatial mesh. Finer meshes allow sharper gradients to be resolved more accurately but increase runtime and memory usage.
19. Set the absolute and relative error tolerances for the solver. The relative tolerance controls the allowed error relative to the local solution magnitude. The absolute tolerance controls the allowed error in absolute magnitude.

***Note:*** The 1D solver uses MATLAB’s bvp4c function to solve the resulting linear or nonlinear boundary value problem. The mesh-point parameter controls spatial resolution, while the solver tolerances determine when the numerical solution is sufficiently accurate.

***Note:*** Fewer mesh points reduce memory usage and compute time but may be insufficient to resolve steep gradients.

17. Open the Solver tab (Figure 9) and configure the numerical solver settings.

20. For 2D problems, set the upper bound for the cell-centered finite-volume discretization mesh in the *x*- and *y*-directions. Increasing the mesh resolution improves spatial accuracy but increases memory usage and runtime.

***Note:*** In 2D the solver strategy depends on the reaction term. *Linear* reaction-diffusion is solved using an iterative linear solver (GMRES). *Nonlinear* reaction terms are solved using pseudo-time integration with ode15s, meaning the system is numerically evolved in an artificial time variable until it relaxes to a steady state. Most users can start with the default settings and adjust them only if convergence is slow or fails.

21. For linear problems (using GMRES), specify the following parameters (or use the default values):
  a. absolute tolerance: how small the remaining numerical error must be before the linear solver is considered converged.
  b. restart: how many solver iterations are performed before the internal approximation is reset. Larger values can enable convergence for difficult problems but increase memory usage.
  c. maximum cycles: maximum number of restart cycles the solver is allowed to perform before stopping. Larger values may help with convergence problems but can increase the runtime.
22. For nonlinear problems (using ode15s) specify the following parameters (or use default values):
  a. maximum pseudo-time: the maximum artificial integration time allowed for the system to relax to steady state.
    ***Note:*** If the system fails to reach steady state, try increasing the maximum pseudo-time.
  b. relative error tolerance: the permitted relative local error during integration.
  c. absolute error tolerance: the permitted absolute local error during integration.
    ***Note:*** Solver tolerances control the required numerical accuracy. Tighter tolerances increase accuracy but may significantly increase runtime. If convergence fails or the solution oscillates, first increase the maximum mesh resolution, then consider relaxing tolerances.

### Finalize setup and preview all user-defined parameters

**Timing: 2–5 min**

The Preview displays all user-defined settings, including domain geometry, kinetic parameters, introduction of noise, solver tolerances, and custom reaction equations, allowing the verification of input settings before generating the configuration file.

23. Open the Summary tab and select Preview.
24. Optionally run a single simulation by clicking Run to verify that the chosen parameters and equations produce sensible results.
25. If necessary, adjust the settings and repeat the test run.
26. Once satisfied, save the configuration by clicking Save & Close.

***Note:*** The configuration is stored as a standalone .mat file and can be reused for subsequent simulations. Detailed documentation of the configuration structure is provided in the associated Git repository.

### Running simulations

**Timing: Variable; depending on user settings, number of simulation runs, and system specifications, this step can take from minutes for small 1D simulations to days for large, finely resolved 2D parameter screens**

In this step, we describe how to run stochastic reaction-diffusion simulations using a previously saved configuration file (cfg). All simulations reported in this protocol were run on a MacBook Pro laptop computer with an Apple M2 chip and 16 GB of memory. Large-scale 2D parameter screens may benefit from execution on a multicore workstation or cluster compute infrastructure with MATLAB installed.

27. Execute simulations using the saved cfg using one of the following two functions:
  a. result = morphograd.solve.solve_rd_1d(cfg, verbose) for one-dimensional simulations
  b. result = morphograd.solve.solve_rd_2d(cfg, verbose) for two-dimensional simulations

***Note:*** The optional argument verbose is a Boolean variable (0/1 or false/true) that controls whether solver progress and diagnostic information are printed to the console.

28. Execute the solver repeatedly to generate an ensemble of stochastic gradients, using custom MATLAB scripts.

***Note:*** Such scripts typically call the solver multiple times with the same configuration file and collect outputs across runs for subsequent analysis.

29. Collect simulation outputs across runs in a suitable data structure for downstream analysis, for example a MATLAB table or matrix.

**CRITICAL:** Each simulation corresponds to a single stochastic realization of the morphogen gradient. Statistical quantities such as positional error and average readout positions require ensembles of independent simulations.

***Note:*** Each simulation returns a MATLAB struct result which organizes outputs into substructures. This design separates numerical solutions, geometric information, model parameters, diagnostics, and boundary conditions. A detailed description of the simulation outputs and data structures is available in the Git documentation associated with this protocol.

***Note:*** For reproducibility and downstream use, simulation can optionally be exported to standard file formats such as .csv.

## Expected outcomes

**Figure 10:**
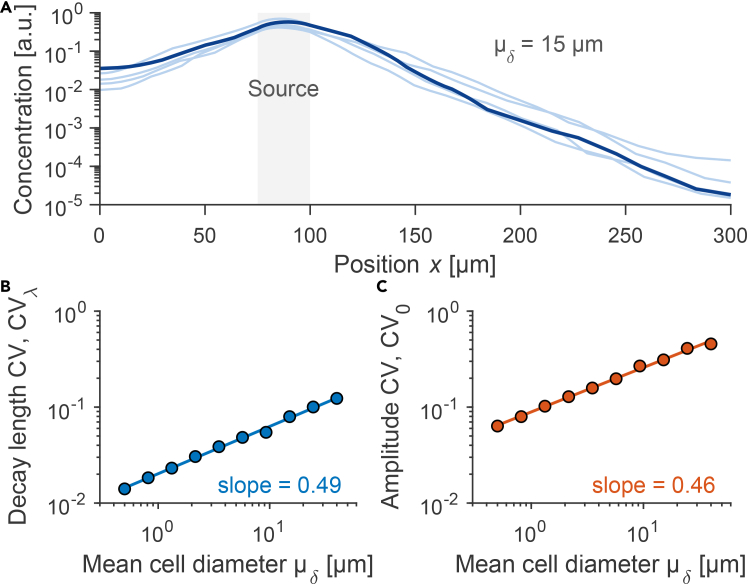
Representative 1D stochastic simulation outputs and example parameter screen analysis (A) Five noisy gradients for one representative mean cell diameter. (B, C) Analysis of how decay length variability and amplitude variability scale with cell size. Each data point is based on 200 independent simulation runs. Error bars indicate SEM and are smaller than the symbols.

**Figure 11:**
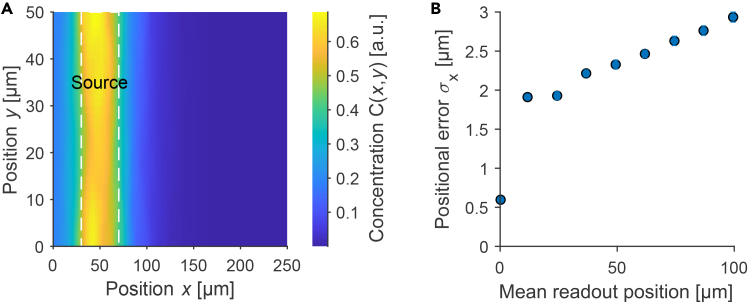
Representative 2D stochastic simulation output and example analysis (A) Simulated 2D concentration field. (B) Scaling of the positional error with the readout position. Each data point is based on 200 independent simulation runs. Error bars indicate SEM.

Successful execution of this protocol yields simulated stochastic morphogen gradients in one- and two-dimensional cellular tissues (Figures 10A and 11A). The framework produces ensembles of noisy concentration profiles from which key quantities, such as amplitude, decay length variability, and positional error can be extracted. These results provide quantitative insight into how variability in cellular parameters influences tissue patterning precision.

## Quantification and statistical analysis

Morphogen gradient precision is quantified using ensembles of independent stochastic gradient simulations. Simulation outputs are analyzed to assess how variability in morphogen gradient profiles translates into variability in patterning outcomes.

One approach to quantifying precision is to define a readout position, which corresponds to the spatial location at which a concentration threshold is crossed and marks the boundary between different cell fates. Variability in this readout position across independent stochastic gradient realizations is quantified by the positional error, defined as the standard deviation of the readout positions, and is a measure of boundary precision.

More generally, summary statistics can be used to quantify the variability across stochastic gradient simulations. These may include, for example, the mean readout position, the positional error, coefficients of variation, and corresponding standard errors. Together, these measures allow the assessment of gradient robustness and precision across ensembles of stochastic realizations. Figures 10B and 10C, and 11B show representative examples of such analyses, but they are not exhaustive. Additional workflows for stochastic gradient simulations, parameter screens, parallelization, reproduction of published figures, and further analysis and visualization methods are provided in the associated Git repository.

## Limitations

This framework models stochastic morphogen gradients in 1D and 2D tissues, assuming a steady state and independent cell-to-cell variability. It does not explicitly simulate stochastic reaction or diffusion events over time. Instead, variability is represented through independently sampled parameters for each cell. The framework can therefore be used to study how cell-to-cell differences, whether intrinsic or extrinsic in origin, affect patterning precision, but it does not model the underlying stochastic process itself. The framework does not capture dynamic tissue growth, feedback from gene regulatory networks, or spatial correlations in kinetic parameters. Molecular-scale processes such as receptor trafficking are neglected. In addition, cell shapes are either approximated as line segments (1D) or as rectangles (2D). Consequently, results are best interpreted as simplified epithelial systems that have reached growth termination and are in a steady state. Simulations may become computationally demanding for large 2D domains.

## Troubleshooting

### Problem 1

Geometry validation error. In 1D, the domain length is not a multiple of the cell diameter. In 2D, the domain length in *x*-direction or *y*-direction is not a multiple of the cell diameter in *x*or *y* (Steps 3a, b).

### Potential solution

• Set the domain lengths to be whole multiples of the respective cell diameters.
• Use the grid preview to visually confirm alignment before running the solver.

### Problem 2

The 1D solver does not converge, bvp4c fails, or returns a large residual. The solution appears unphysical (Steps 14**–**16 or 18**–**19).

### Potential solution

• Improve the initial guess: use a preset initial guess, or define a smooth, monotonic profile consistent with production in the source region and decay in the patterning domain (Steps 14**–**16).
• Increase the maximum number of mesh points allowed for bvp4c in the Solver tab (Step 18).
• Relax solver tolerances (RelTol/AbsTol) temporarily to diagnose if the failure is numerical or model-driven (Step 19).

### Problem 3

The 2D solver does not reach steady state within the maximum pseudo-time (ode15s stops early), that is, the reported pseudo-time reaches its maximum, and the solver terminates before convergence (Steps 20**–**22).

### Potential solution

• Increase mesh resolution in *x*- and *y*-directions if steep gradients are expected (Step 20).
• Increase the maximum pseudo-time to allow the system to relax to steady state (Step 22a).
• Check if boundary conditions are compatible with a steady-state solution.
• If the reaction term is highly nonlinear or stiff, consider adjusting solver tolerances (Steps 22 b, c).

### Problem 4

The solution contains negative concentrations or non-physical (e.g., complex) values (Steps 10 and 14**–**16).

### Potential solution

• Verify that parameter sampling does not generate extreme values. High CVs can produce very large or very small parameters under a log-normal distribution.
• Reduce CV values for the most sensitive parameters (Step 10).
• Use a non-negative initial guess (Steps 14**–**16).
• After stability is confirmed, tighten solver tolerances if needed (Steps 19, 21**–**22).

### Problem 5

The gradient is nearly flat, or no gradient forms (Steps 3, 6, and 12).

### Potential solution

• Verify that the source region is correctly defined in the Domain tab (Step 3).
• Ensure that the reaction term correctly uses *H* to restrict production to the source domain (Step 6).
• Check that boundary conditions are not forcing a constant solution, for example through identical Dirichlet values on all boundaries (Step 12).

### Problem 6

The gradient is very steep, with clear numerical artifacts (Steps 18**–**22).

### Potential solution

• Increase the maximum mesh resolution (Steps 18, 20).
• Tighten error tolerances after sufficient spatial resolution is reached (Steps 19, 21a, 22 b).

### Problem 7

Runs are very slow, exceed memory limits (especially for large 2D domains), or MATLAB becomes unresponsive (Steps 3, 10, 18, and 20).

### Potential solution

• For large 2D domains, avoid unnecessarily fine grids during exploration and initially start out with a smaller domain (Step 3).
• Reduce the maximum number of mesh points in *x*- and *y*-direction in 2D or maximum mesh points in 1D, to test settings quickly before scaling up (Step 18, 20).
• Reduce CV values if extreme parameter sampling leads to stiff dynamics that require tiny solver increments (Step 10).

### Problem 8

Parallel scripts fail to start, or errors related to parfor occur.

***Note:*** This problem relates to the MATLAB environment outside of the MorphoGrad GUI.

### Potential solution

• Reduce the number of workers nWorkers to match available resources.
• Verify that the Parallel Computing Toolbox is installed, by typing ver into MATLAB’s Command Window. This command lists all available toolboxes. If the toolbox is not available, try installing it.
• Ensure that variables written inside parfor are preallocated and indexed only by the loop variable.

## Resource availability

### Lead contact

Further information and requests for resources and reagents should be directed to and will be fulfilled by the lead contact, Dagmar Iber (dagmar.iber@bsse.ethz.ch).

### Technical contact

Technical questions on executing this protocol should be directed to and will be answered by the technical contact, Jan A. Adelmann (jan.adelmann@gmail.com).

### Materials availability

This study did not generate new unique materials.

### Data and code availability

All original code has been deposited in the GitLab repository (https://git.bsse.ethz.ch/iber/Publications/2026_adelmann_morphograd) and is publicly available. The DOI of an archived version of the repository is listed in the key resources table.

## Acknowledgments

This work was partially funded by the 10.13039/501100001711Swiss National Science Foundation through grant 315230_219990 (to D.I.).

## Author contributions

J.A.A.: conceptualization, methodology, software, validation, formal analysis, visualization, and writing. R.V. and D.I.: conceptualization, supervision, and writing.

## Declaration of interests

The authors declare no competing interests.

## Declaration of generative AI and AI-assisted technologies in the writing process

During the manuscript preparation, the authors used ChatGPT (OpenAI) for language refinement and stylistic editing. All AI-assisted text was reviewed and edited by the authors, who take full responsibility for the content of the manuscript.
